# Supplementary material for: Comprehensive Analysis of the INDETERMINATE DOMAIN (IDD) Gene Family and Their Response to Abiotic Stress in Zea mays
Source: Int J Mol Sci. 2023 Mar 24;24(7):6185. doi: 10.3390/ijms24076185 (PMC10094743; doi:10.3390/ijms24076185)
Supplement: Supplementary file 1 [file ijms-24-06185-s001.zip › Feng et al Figure S1-S3.pdf]

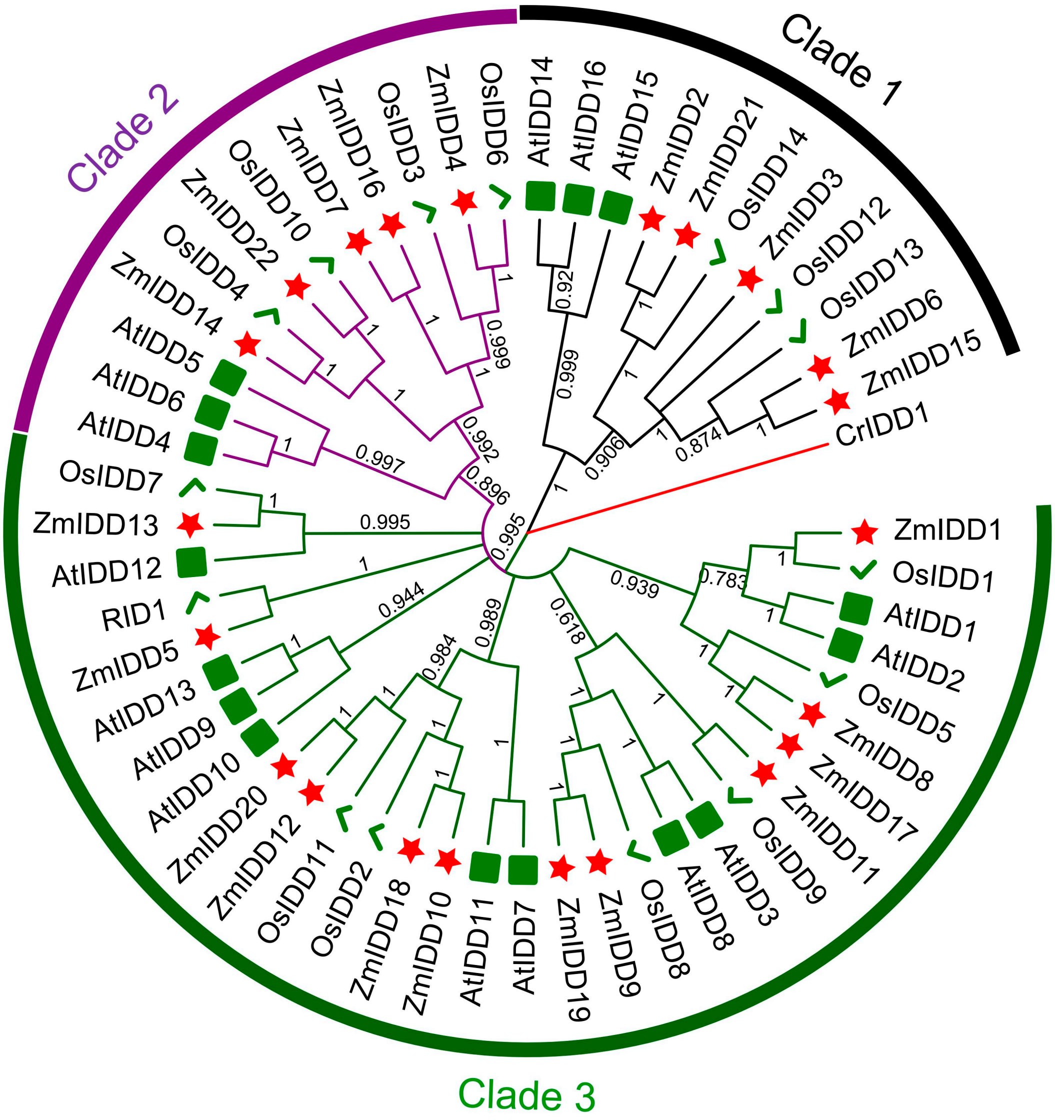

Figure S1. Phylogenetic tree of full-length ZmIDD, AtIDD and OsIDD proteins using the Minimum Evolution method. The optimal tree with the sum of branch length = 3.31299193 is shown. The analysis involved 54 amino acid sequences. All positions containing gaps and missing data were eliminated. There were a total of 94 positions in the final dataset. The different colored arcs indicate subfamilies of the IDD proteins. Different colour shapes represent IDDs from maize (☆), rice (√), and Arabidopsis (□). IDD in *Chlamydomonas reinhardtii* was selected as outgroup.

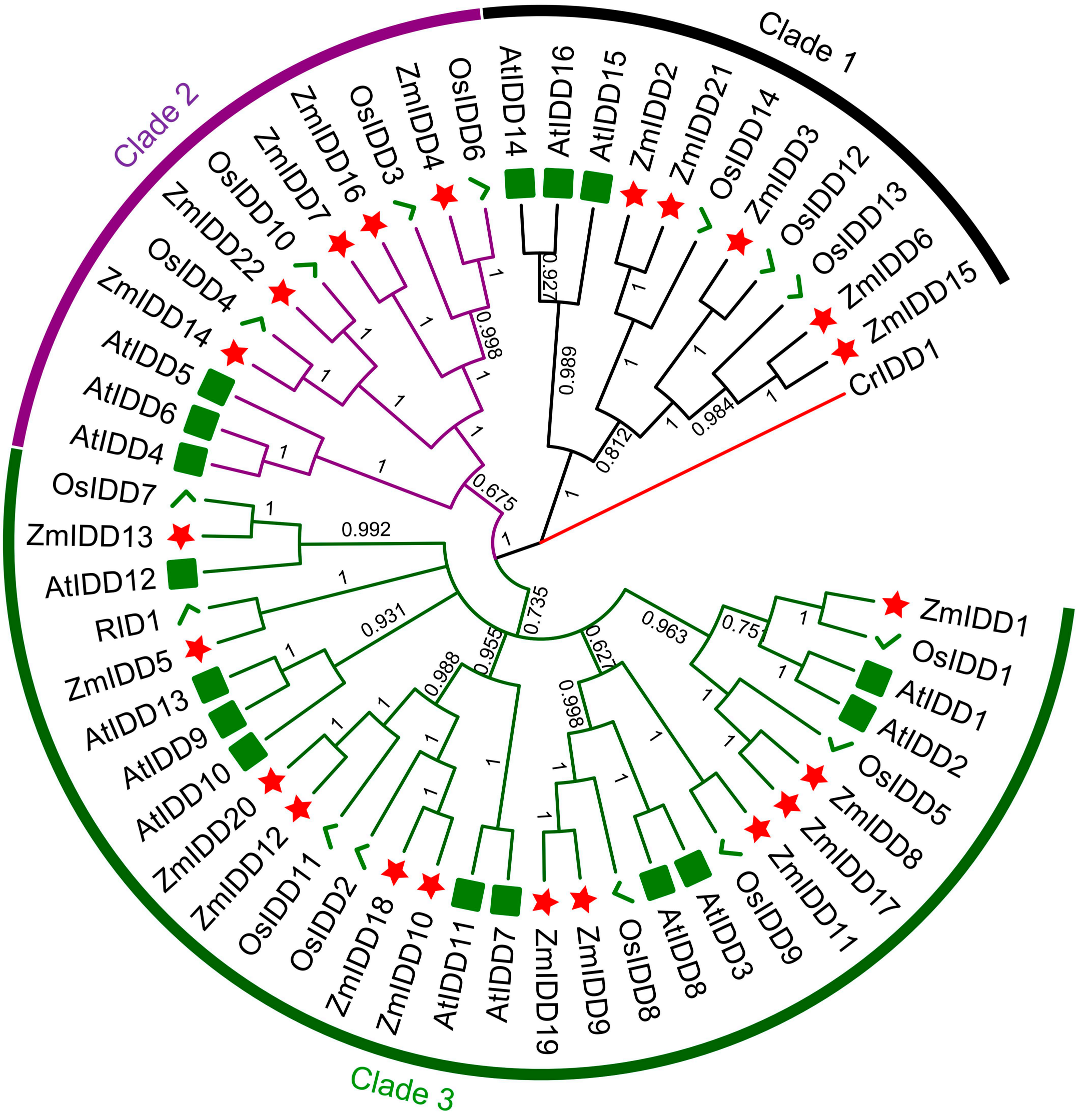

Figure S2. Phylogenetic tree of full-length ZmIDD, AtIDD and OsIDD proteins using the Neighbor-Joining method. The optimal tree with the sum of branch length = 3.31299193 is shown. The analysis involved 54 amino acid sequences. All positions containing gaps and missing data were eliminated. There were a total of 94 positions in the final dataset. The different colored arcs indicate subfamilies of the IDD proteins. Different colour shapes represent IDDs from maize (☆), rice (√), and Arabidopsis (□). IDD in *Chlamydomonas reinhardtii* was selected as outgroup.

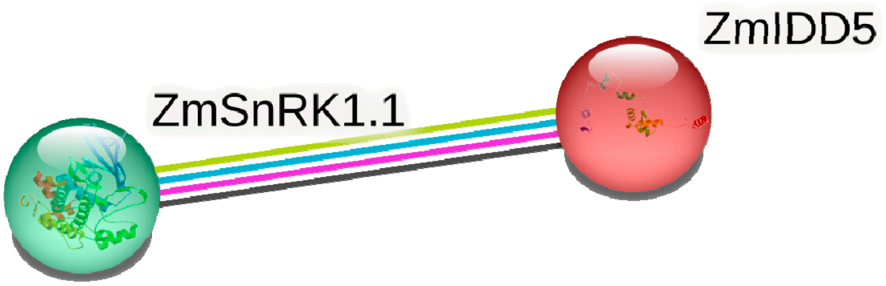

Figure S3. Interaction networks between ZmSnRK and ZmIDD proteins.
